# Patient Perspectives on Quantified Self Technologies and Healthcare Costs Among Patients with Diabetes in Zimbabwe

**DOI:** 10.3390/ijerph23050663

**Published:** 2026-05-18

**Authors:** Belinda Mutunhu, Baldreck Chipangura

**Affiliations:** Department of Information Systems, School of Computing, College of Science, Engineering and Technology, University of South Africa, Pretoria 0003, South Africa; chipab@unisa.ac.za

**Keywords:** quantified self-technologies, digital health, diabetes management, healthcare costs, affordability, socio-economic determinants, public health, low and middle-income countries, Zimbabwe

## Abstract

**Highlights:**

**Public health relevance—How does this work relate to a public health issue?**

- Examines how individuals with diabetes in Zimbabwe experience the financial implications of adopting quantified self technologies for disease management.
- It addresses digital health affordability within the broader context of chronic disease management in resource-constrained settings.

**Public health significance—Why is this work of significance to public health?**

- Findings indicate that technology-related costs extend beyond device acquisition to include recurring digital expenses, infrastructural reliability, and continued reliance on conventional healthcare services.
- The study highlights how socio-economic vulnerability shapes the sustainability of digital health engagement at the household level.

**Public health implications—What are the key implications or messages for practitioners, policy makers and/or researchers in public health?**

- Digital health initiatives in similar contexts may benefit from incorporating affordability and infrastructural readiness considerations into implementation planning.
- Patient-level cost experiences should inform broader discussions on equitable and context-sensitive digital health deployment.

**Abstract:**

The growing use of quantified self-technologies (QST) in chronic disease management is linked to better self-monitoring and patient engagement. However, little is known about how patients in resource-constrained settings fund and sustain the use of QST in diabetes self-management. This study asked: “How do patients with diabetes perceive and experience the economic burden of using QST in Zimbabwe?” Using a qualitative design, 20 patients with diabetes participated in semi-structured interviews. The reflexive thematic analysis of Braun and Clarke generated three interrelated themes: technology investment costs, conventional healthcare costs, and socio-economic constraints. The findings show that the economic experience of QST adoption is context-dependent and is shaped by the financial realities of patients with diabetes and their access to technology. By focusing on patient-level cost experiences, the study adds qualitative evidence to public health debates on digital health affordability and highlights the need to assess perceived financial implications within a third-world socio-economic context. It is concluded that, although QST is available in third-world countries, sustained use depends on the financial capacity of patients with diabetes.

## 1. Introduction

Diabetes mellitus represents an increasing global public health challenge and a leading contributor to long-term healthcare expenditure, particularly in low and middle-income countries (LMICs) [1,2,3,4]. Effective management requires sustained self-monitoring, medication adherence, dietary regulation, and routine clinical consultations, generating continuous financial pressure on both households and health systems [5,6]. In sub-Saharan Africa, the burden is compounded by late diagnosis and costly complications such as cardiovascular disease, renal failure, and lower limb amputations, which significantly increase hospitalisation and treatment costs [7,8]. In Zimbabwe, diabetes-related hospitalisation costs rank among the highest in the region, and insulin affordability remains a major barrier to effective disease control [9,10,11]. These realities position diabetes not only as a clinical condition but also as a persistent economic strain within fragile health systems.

In response to the growing challenge of non-communicable diseases and the shift to patient-centred care, quantified self-technologies (QST), consisting of wearable fitness trackers, mobile health applications, and continuous glucose monitor (CGM) systems, have become prominent digital health technologies for managing chronic diseases [12,13]. The use of QST enables real-time monitoring of physiological and behavioural data. The potential benefits of QST include better glycaemic control, improved treatment compliance, and increased patient engagement with self-management [14,15]. Within dominant health discourse, the benefits of QST are frequently related to improved self-health management and long-term economic benefits [16,17,18].

The relationship between clinical improvement and economic sustainability remains context dependent. Evidence from high-income settings suggests potential long-term cost reductions [18,19], but these results typically arise in systems with subsidised technology, established healthcare infrastructure, and robust health information technology [17,20]. Emerging studies from middle-income settings, including Gulf countries, show positive patient-reported outcomes with digital diabetes management, but do not consistently show reduced out-of-pocket spending or better affordability [21,22]. This suggests that improved health monitoring does not automatically lessen households’ financial burden.

In LMIC contexts, patients frequently bear the full costs of device acquisition, internet connectivity, electricity, maintenance, and subscription services, in addition to ongoing consultation, laboratory, and medication expenses [21,22,23]. Under such conditions, the assumption that QST adoption necessarily reduces personal healthcare expenditure warrants closer examination [24,25]. Although the clinical effectiveness of digital self-monitoring tools has been widely documented, their economic implications from the patient perspective remain comparatively underexplored in third-world contexts, particularly in African public health systems [26,27].

Zimbabwe provides a critical case for such analysis. Diabetes care in the country is characterised by high out-of-pocket spending, limited insurance coverage, medication supply instability, and infrastructural fragility [9,10,11,28]. These structural constraints shape how digital health interventions are accessed, financed, and sustained. Consequently, assessing the economic experiences of patients who adopt QST within this environment offers important insights into the broader question of digital health affordability in resource-constrained settings.

Against this backdrop, this study addresses the limited attention given to patient-level cost experiences in digital diabetes self-management within resource-constrained settings. Accordingly, the aim of this study is to explore how patients with diabetes in Zimbabwe experience the costs associated with adopting quantified self-technologies within their everyday healthcare practices. The study is guided by the following research question: In what ways do patients with diabetes perceive and experience the economic burden associated with the use of quantified self-technologies in Zimbabwe? By foregrounding lived cost realities rather than assuming economic benefits, this research contributes context-sensitive evidence to ongoing public health discussions on the affordability and sustainability of digital health interventions in low-resource settings.

## 2. Literature Review

### 2.1. Economic Burden and Out-of-Pocket Financing in Diabetes Care

The economic burden of diabetes is well documented across both high- and low-income countries [2,3]. In LMICs, however, this burden is disproportionately borne by households due to high levels of out-of-pocket expenditure and limited insurance coverage [10,11]. Chronic disease management entails recurring costs related to medication, laboratory testing, clinical consultations, and complication management [5,6].

Evidence from sub-Saharan Africa indicates that hospitalisations related to diabetes ailments, such as foot infections, renal complications, and amputations, significantly increase long-term expenditure [7,8]. Country-specific estimates demonstrate substantial variation in annual per-patient costs, reflecting differences in infrastructure, pricing, and health system financing models [2,28,29,30]. These cost dynamics have intensified interest in digital health tools positioned as mechanisms for supporting preventive self-management and potentially moderating downstream healthcare expenditure.

### 2.2. Quantified Self Technologies and the Cost-Reduction Narrative

Quantified self-technologies, including wearable sensors, continuous glucose monitoring systems, and mobile health applications, have been associated with improved glycaemic control, behavioural adherence, and patient engagement [31,32,33,34]. Within digital health discourse, these improvements are frequently linked to expectations of reduced hospitalisations and fewer emergency interventions [17,18].

This cost-reduction narrative rests on several assumptions: that early detection through continuous monitoring prevents complications [34,35]; that behavioural tracking supports sustained lifestyle modification [18,19] and that remote engagement may reduce in-person consultations [35,36,37,38]. Empirical studies conducted in digitally mature health systems report improvements in glycaemic outcomes, with some suggesting downstream efficiencies [33,34,35].

However, much of this evidence emerges from contexts characterised by stable infrastructure, institutional integration, and partial or full technology subsidisation [17,20,36,39]. As such, the transferability of these findings to settings in which patients directly bear technology and connectivity costs remains uncertain.

### 2.3. Structural Constraints and Digital Health Adoption in LMICs

In LMICs, the implementation of digital health technologies occurs within infrastructural and economic environments that differ substantially from high-income contexts [21,22,23,40,41]. Device affordability, connectivity instability, electricity reliability, technical support availability, and digital literacy have all been identified as critical determinants of adoption and sustained use [22,23,41,42].

Some studies suggest that technology adoption may introduce additional monitoring or maintenance costs rather than reducing overall expenditure [39,40,41]. For example, comparative analyses have reported no statistically significant reductions in healthcare spending among users of self-monitoring technologies relative to non-users in certain contexts [37]. These findings challenge the assumption that digital self-tracking inherently reduces patient-level costs.

Collectively, existing research indicates that the economic implications of digital health adoption are highly context-dependent and shaped by structural conditions beyond the technology itself.

### 2.4. Evidence Gap and Research Imperative

Despite increasing digital health deployment in African contexts, empirical investigation into the patient-level cost consequences of QST remains limited [26,27,42,43]. While clinical and behavioural outcomes are frequently assessed, fewer studies examine how device acquisition, recurring connectivity expenses, maintenance costs, and broader socio-economic pressures shape affordability.

Moreover, much of the literature implicitly assumes stable access to infrastructure and uninterrupted digital connectivity—conditions that are not uniformly present in many LMIC settings [21,23]. This creates a disconnect between theoretical projections of cost moderation and the lived financial realities of patients operating within resource-constrained environments.

Accordingly, there is a need for context-sensitive, patient-centred research that examines how QST intersects with existing cost structures in everyday chronic disease management. The present study addresses this gap by investigating how patients with diabetes in Zimbabwe experience the costs associated with adopting QST.

## 3. Theoretical Framework Underpinning the Study

This study draws on the Theory of Planned Behaviour (TPB) [44], the Unified Theory of Acceptance and Use of Technology (UTAUT) [45], and Social Cognitive Theory (SCT) [46] as complementary sensitising frameworks to interpret how patients navigate the costs associated with QST. Rather than functioning as predictive models, these theories provided structured lenses for understanding how individual beliefs, social influences, and environmental constraints shape technology use within resource-constrained settings.

### 3.1. Theory of Planned Behaviour (TPB)

The Theory of Planned Behaviour [44] posits that behaviour is shaped by attitudes, subjective norms, and perceived behavioural control. Within this study, TPB informed the examination of how patients evaluate QST in relation to cost (attitudes), how social expectations from family and healthcare providers influence adoption decisions (subjective norms), and how financial and infrastructural constraints affect their perceived ability to sustain use (perceived behavioural control). The construct of perceived behavioural control was particularly relevant to the analysis of how affordability concerns influenced sustained engagement with digital self-monitoring tools.

### 3.2. Unified Theory of Acceptance and Use of Technology (UTAUT)

UTAUT [45] conceptualises technology adoption through performance expectancy, effort expectancy, social influence, and facilitating conditions. In this study, UTAUT supported the exploration of how patients’ expectations regarding health improvement intersected with cost considerations (performance expectancy), how usability influenced continued engagement (effort expectancy), and how infrastructural conditions—such as device affordability, internet access, electricity reliability, and technical support—shaped sustained use (facilitating conditions). The framework was particularly useful in analysing how enabling conditions moderated the perceived financial viability of QST adoption.

### 3.3. The Social Cognitive Theory (SCT)

Social Cognitive Theory [46] emphasises the reciprocal interaction between personal factors, environmental conditions, and behaviour. SCT informed the analysis of how environmental constraints such as unemployment, food insecurity, and infrastructure instability interacted with individual self-efficacy and outcome expectations regarding QST use. The concept of self-efficacy was relevant in understanding how patients assessed their ability to manage diabetes in the presence of ongoing technology-related costs

Rather than functioning as competing explanatory models, TPB, UTAUT, and SCT were used in an integrative manner to guide interview design and thematic interpretation. Each framework contributed distinct analytical sensitivity: TPB illuminated attitudinal and control dimensions; UTAUT structured analysis of adoption and enabling conditions; and SCT contextualised behavioural decisions within broader socio-economic environments. This combined approach allowed the study to interpret patient-reported cost experiences without extending beyond the empirical scope of the data.

## 4. Methodology

### 4.1. Research Approach

This study adopted a qualitative exploratory research design informed by phenomenological principles [47,48]. The approach was selected to enable an in-depth examination of how patients with diabetes experience and interpret the costs associated with adopting quantified self technologies within their everyday healthcare practices. A qualitative design was considered appropriate given the study’s focus on subjective meanings, lived financial realities, and context-specific constraints rather than measurable economic outcomes [49].

While not conducted as a strict transcendental phenomenological study, the research drew on phenomenological sensibilities to foreground participants’ experiential accounts of technology use, affordability, and health management within Zimbabwe’s public health context. This interpretive orientation enabled the study to examine how cost perceptions are shaped by socioeconomic circumstances, infrastructure conditions, and personal coping strategies.

### 4.2. Sampling and Participant Selection

Purposive sampling [48] served as the primary recruitment strategy for this study. Participants were intentionally selected based on their direct experience using QST for diabetes self-management within the Zimbabwean healthcare context. This approach enabled the inclusion of individuals who could provide detailed accounts of technology-related cost experiences.

To be eligible, participants were required to: (1) be aged 18 years or older; (2) have a confirmed diagnosis of diabetes; (3) have used at least one QST device or mobile health application for a minimum of six months; and (4) demonstrate sufficient digital literacy to engage with the technology independently. These criteria ensured that participants maintained sustained experiential familiarity with QST and its associated costs.

Given the absence of a formal registry of QST users in Zimbabwe and the dispersed nature of adoption, snowball sampling was employed as a supplementary strategy. Participants were invited to refer other eligible individuals within their networks. All referred participants were screened using the same inclusion criteria to ensure consistency. Recruitment occurred through multiple entry points, including clinic-based referrals and diabetes support networks, to enhance experiential variation and minimise homogeneity.

### 4.3. Sample Size and Data Saturation

In this study, data saturation from the analysis of interview transcripts determined the sample size. Data saturation was confirmed after analysing 20 transcripts from the interviews. Data collection and analysis proceeded concurrently, allowing for continuous evaluation of emerging themes. Data saturation was achieved when analysis of successive interview transcripts yielded no new first-order codes and when thematic patterns became conceptually stable across participants. Saturation was observed after the eighteenth interview, and two additional interviews were conducted to confirm analytical redundancy and strengthen interpretive confidence. In qualitative research, sample size is determined by analytical depth and thematic sufficiency rather than statistical representativeness [50].

### 4.4. Participant Characteristics

From the sample of 20 transcripts that were analysed until the saturation point, there were 12 (60%) female and 8 (40%) male participants. Additionally, 16 (80%) were employed, 2 (10%) were students, and 2 (10%) were unemployed. The age distribution was as follows: 18–35 years (7 (35%)); 36–45 years (5 (25%)); 46–65 years (8 (40%)).

### 4.5. Interview Questions

Semi-structured interview questions were used to explore the perceptions of patients with diabetes. The interview questions were informed by the constructs of the theoretical framework of this study. Table 1 provides a mapping of the interview questions against the theoretical framework constructs.

### 4.6. Interview Procedure

Interviews were conducted face-to-face or through Microsoft Teams based on participant preference. The duration of an interview was approximately 45 min. Ethical requirements were followed during data collection, which included obtaining consent for participation, audio verbatim recording and transcription. Field notes were taken during and immediately after each interview to capture non-verbal cues and the researcher’s reflections. Confidentiality was maintained by anonymizing all participant data.

Given that this study examines digital access constraints, we recognise that participants who engaged in online interviews may have had relatively better connectivity or digital familiarity than those interviewed face-to-face. While both modalities followed the same semi-structured guide, the potential influence of digital access on participation was considered reflexively during analysis. This limitation is acknowledged as a possible source of variation in the articulation of technology experiences.

### 4.7. Data Analysis

Data analysis followed the reflexive thematic analysis proposed by Braun and Clarke [50]. This approach enabled systematic identification of patterned meanings across participant accounts while maintaining sensitivity to contextual and interpretive dimensions of the data.

The interview transcripts were analysed using ATLAS.ti version 25.0.1.32924 software to facilitate structured coding and data management. During the familiarisation phase, transcripts were read multiple times to achieve immersion in the data, and reflexive memos were recorded to document preliminary analytic observations and emerging insights.

Initial coding was conducted inductively, focusing on meaningful segments of text concerning technology acquisition, recurring financial obligations, infrastructural constraints, and socio-economic vulnerabilities. This phase generated 46 first-order codes derived directly from participants’ narratives. Coding was undertaken independently by the primary researcher and subsequently reviewed collaboratively with a co-author who served as a second coder. Rather than calculating statistical inter-rater reliability, coding discussions were used to refine code definitions, clarify conceptual boundaries, and resolve interpretive discrepancies. Where differences arose, transcripts were revisited to ensure that coding decisions remained grounded in participant accounts.

Through iterative comparison and clustering of conceptually related codes, 14 preliminary thematic groupings were identified. These groupings were further refined through recursive engagement with the full dataset, yielding 4 overarching analytical themes that captured the dominant cost dynamics associated with the adoption of quantified self-technology. Themes were reviewed to ensure internal coherence, conceptual distinctiveness, and representation across multiple participant accounts rather than reliance on isolated quotations.

Consistent with reflexive thematic analysis, coding decisions, theme refinements, and analytic memos were systematically documented throughout the process, enabling a transparent trace of the progression from raw data to the final thematic structure. The theoretical frameworks (TPB, UTAUT, and SCT) informed interpretation during later analytic stages but were not imposed during initial coding, thereby preserving the inductive integrity of the analysis.

### 4.8. Trustworthiness and Rigour

To enhance credibility, dependability, and confirmability, several strategies were employed. First, independent coding between the primary researcher and co-author strengthened interpretive robustness and reduced individual bias. Coding discrepancies were discussed until consensus was reached.

Second, a detailed audit trail was maintained throughout the research process, documenting coding decisions, theme refinement, and analytic memos. This process enhanced transparency and enabled systematic tracking of interpretive development.

Third, reflexive memoing was used during data familiarisation and theme construction to acknowledge the researchers’ interpretive role and minimise unexamined assumptions.

Finally, thick description, through the inclusion of verbatim quotations, was employed to ensure that thematic claims remained grounded in participants’ narratives and to allow readers to evaluate interpretive plausibility.

## 5. Results

Reflexive thematic analysis of the interview data generated three overarching themes describing how participants experienced the costs associated with adopting QST in diabetes management. The themes were as follows: technology investment costs; conventional healthcare costs; socio-economic constraints.

Each theme captures distinct but interrelated dimensions of participants’ reported cost experiences. The findings are presented below with illustrative quotations to foreground participant accounts.

### 5.1. Theme 1: Technology Investment Costs

Data analysis found that the initial cost for using QST for managing diabetes was determined by the cost of a QST-compatible device. On the other hand, the ongoing costs were determined by the broadband data cost, subscription fees, technical support cost and electricity cost (power supply). The following section presents the results under the subthemes.

#### 5.1.1. Cost of the Device

Data analysis revealed that 15 patients already owned smartphones, which was a prerequisite for utilising QST. Therefore, the cost of purchasing a device was not an impediment. Furthermore, these participants perceived the technology as beneficial, regarding the cost of devices as a justifiable, singular investment in their long-term health management.

*“I already owned a smartphone, so I did not need to buy a new one.”*

(P2)

*“I needed to upgrade my smartphone. However, it was costly. I know it is a one-time purchase, and the phone will last me for years. I was willing to invest in it as a means to regulate my glucose levels and prevent more significant issues.”*

(P7)

Some individuals with diabetes found the upfront cost of a QST-compatible device a major financial strain. For those without one, buying the device meant reallocating already limited funds. To afford it, some took extra work, borrowed money, or received financial help from family. Participants P12 and P14 noted,

*“I needed to own a smartphone, but the prices demanded for these smartphones and smartwatches are excessively high and I cannot afford them.”*

(P12)

*“I did not have a smartphone, so I had to do piece jobs to earn money to buy a smartphone, and I bought it together with a power bank in case there was no electricity.”*

(P14)

Quotations from P12 and P14 indicate that people with diabetes lacked equal access to QST-compatible devices. Thus, adopting QST depended not only on perceived health benefits but also on immediate affordability.

#### 5.1.2. Broadband Data Costs

Broadband data costs were a barrier to sustained QST use. Unlike the one-time expense of acquiring a device, internet access required ongoing payment. Participants reported that a continuous connection was essential for uploading readings, receiving notifications, and communicating with healthcare providers. Many participants revealed that, because they found broadband expensive, they used data-saving strategies such as delaying uploads or intermittently stopping app use when data ran out. Participants P3, P8, and P14 noted,

*“As for data, it is expensive, although I sacrifice buying it regularly to access my app. The support that I need is a constant data supply, because I usually lack money to purchase the data bundles.”*

(P3)

*“I would love to have data bundles to use this application, but they are expensive.”*

(P8)

*“To effectively use this app, I need constant access to the internet, access to prepaid Wi-Fi, preferably for the whole month. This might actually save me from worrying about money when purchasing broadband data. I’m a student, I do not have a stable source of income, and sometimes I run out of data bundles.”*

(P14)

These accounts illustrate how recurring data bundle costs transform QST from supportive self-management tools into financially unstable health practices. Although participants recognised the potential benefits of continuous data connectivity, the unpredictable and recurrent nature of data expenses forced them to ration usage, resulting in irregular engagement with the technology. Thus, continued QST use depended on a person’s ability to fund broadband access.

#### 5.1.3. Subscription Fees

Subscription fees were an unexpected cost for individuals with diabetes. Participants revealed that the QST application they used required monthly or annual subscriptions to activate certain features, including medication reminders, personalised insights, and detailed analytics. For several participants, these recurring fees were reported to add to the cost of the device and internet access. To manage this, patients with diabetes highlighted that they used various strategies, such as relying on donations from family members or subscribing only to essential features they could afford. In this respect, participants P5, P6 and P11 noted,

*“One cousin of mine opted to pay the subscription on the reminders to take medication for me so that I can access and use the app at any given time because I cannot afford. All the subscriptions are catered for by my cousin, who happens to be in the diaspora.”*

(P6)

*“When I was still using Strava before I stopped using it, I had to pay a subscription of twelve dollars a month, which was beyond my reach.”*

(P5)

*“…Another thing is the use of reminders. I do not get to use it often because this feature is paid for, and at times, I fail to get money to pay for it. It is a wish that I pay for it because it is very important.”*

(P11)

In contrast to the patients with diabetes who found subscription fees unaffordable, a few participants (15%) were able to pay subscription fees. They perceived the subscription fees as worthwhile and desirable because they gave them access to essential advanced QST functions. In this respect, participants P3 and P7 noted,

*“Even though this app’s reminder is paid for, I made sure that I have money to pay for that reminder, which I felt was important in managing my condition. I chose to pay an annual subscription, ……. ”*

(P3)

*“The subscriptions are around $3 per month and around $30 per year, which is quite affordable.”*

(P7)

The findings reveal that some patients with diabetes are willing to spend on their health as they deem the benefits as outweighing the costs. However, the contrasting narratives underscore how subscription-based technologies such as QST risk excluding economically vulnerable patients from essential self-management functionalities, thereby constraining the equitable and long-term health promotion potential of QST.

#### 5.1.4. Technical Support Cost

Data analysis found that patients with diabetes had difficulties accessing technical assistance when their devices malfunctioned or applications failed. Insufficient support was experienced by many patients with diabetes who felt abandoned and sometimes worthless when they could not overcome some technical challenges. Even though most QST applications provide online support, patients with diabetes felt that the support was not sufficient to provide solutions for hardware failure. Because of the desperation to have their devices fixed, the patients with diabetes revealed that they sought expert technical services from repair shops, which cost money. In the words of participants P9, P17 and P20:

*“When my device had a problem, there was no one here in Zimbabwe to fix it. I had to send it to South Africa, which cost me so much money and time. It was cheaper to just go back to writing everything in a book.”*

(P9)

*“When I experience issues with my app, I lack a support system. I returned to my doctor because I was unable to operate the app and required assistance with my data.”*

(P17)

*“My smartwatch ceased charging abruptly one day.” I visited numerous phone repair establishments. Nevertheless, none possessed the expertise to rectify the issue. It is now merely a nonfunctional timepiece. I am unable to purchase a new one”.*

(P20)

Data analysis uncovered that a device malfunction introduced unexpected financial costs. Repairing devices brought an additional logistical expense, which risked some of the patients with diabetes abandoning the technology and returning to manual tracking methods.

#### 5.1.5. Electricity (Power) Supply

Participants found that frequent power outages affected their ability to charge devices and maintain internet connectivity. Since smartphones, smartwatches, and Wi-Fi routers require electricity, participants highlighted that interruptions in power supply disrupted the continuity of QST use. In this respect, participants P11 and P16 noted,

*“A power inverter would be necessary since we have power cuts almost daily. A powerful inverter can last for about 48 to 72 h, making it easier for me to recharge my phone, but this is expensive.”*

(P11)

*“There is load shedding every time. I need a solar system or generator as backup power to charge my phone and keep my router on for the internet. However, I cannot afford a solar system.”*

(P16)

The prior quoted participants’ narratives indicate that maintaining uninterrupted device functionality sometimes requires additional investments in alternative power sources. If a participant could not afford a power backup device, it meant that consistent engagement with the digital monitoring device was interrupted. Because of an unreliable power supply, there were participants who contemplated using manual recording methods or scheduling clinic visits. Reliable power supply shaped the sustainable, continuous use of QST. On the other hand, an unreliable power supply demanded that patients with diabetes invest in alternative power supply sources.

### 5.2. Theme 2: Conventional Healthcare Service Costs

Data analysis found that even though the patients with diabetes adopted QSTs, they had to continue to abide by conventional health practices for managing diabetes. Although QST enabled self-monitoring and facilitated access to personal health data, participants continued to incur doctor consultation fees, laboratory testing, and medication refills. The results are now presented.

#### 5.2.1. Doctors’ Consultation Fees

Data analysis uncovered that even though the patients with diabetes were using QST for self-monitoring, they continued to consult physicians. While access to QST health data was essential, it could not replace professional medical expertise. The results suggest that QST functioned primarily as a complement to, rather than a substitute for, clinical consultation. It was found that although patients with diabetes used their own resources to capture and share QST data with physicians, they were still charged consultation fees. Consultation fees were charged whether the discussion was online or in person. Hence, the use of QST applications for diabetes management was perceived as an extra cost. In this respect, participants P1 and P19 noted,

*“…every time an appointment is booked for me, it is mandatory to pay the consultation fee… The doctor cannot be replaced, but the app helps me show him what has been going on in his absence.”*

(P1)

*“I continue to consult my doctor regularly because I appreciate their expertise. We need to discuss the app’s findings and underlying causes if my sugar is elevated.”*

(P19)

Even though the patients with diabetes generally agreed that the use of QST was an extra cost, there were participants who appreciated the benefits of QST. They acknowledged that QST provides essential data that enables them to accurately manage their routines, which culminates in reduced hospital visits.

*“I no longer visit the hospital frequently like I used to. I visit the hospital when there is a need and at times I just communicate with the doctor over the phone.”*

(P7)

*“In terms of cutting costs, the app has assisted me. My physician consultations have been very few compared to when I did not have the app. This has cut a lot of costs because being admitted to a hospital comes with many costs.”*

(P8)

Access to real-time data supported more autonomous management, occasionally reducing the frequency of visits. Nonetheless, consultation fees remained a major cost within the broader diabetes management.

#### 5.2.2. Lab Tests and Medication Refills Cost

The data analysis revealed that the use of QST does not eliminate the need for routine laboratory testing or prescription-based medication refills. While the use of QST provided real-time tracking, participants emphasised that digitally captured data cannot replace laboratory blood tests. Participants revealed that although QST supported daily monitoring and medication adherence, the financial obligations associated with laboratory diagnostics and prescribed medication remained unchanged. Participants described these costs as non-negotiable components of diabetes management and independent of digital tool use. In this respect, participants P9 and P16 noted,

*“I still need to visit the hospital after some time just to get my prescription so that I will be able to buy my medication from the pharmacies.”*

(P9)

*“The application prompts me to administer my medication, which is beneficial.” However, it does not reduce the cost of my medication, nor does it provide me with the prescription. I must still pay for the medication and visit the clinic to obtain the prescription documentation.”*

(P16)

Digital tools were recognised as a mechanism to support established clinical practices for diabetes management, but not as a cost-saving strategy.

### 5.3. Theme 3: Socio-Economic Constraints

Broader socio-economic circumstances influenced how patients with diabetes used QST for the daily management of diabetes. The socio-economic factors that shaped the feasibility of using SQT for diabetes management were food security and employment.

#### 5.3.1. Food Security

Putting food on the table came as the primary financial priority amongst patients with diabetes in this study. Ensuring that a family has sustainable food reserves took precedence over costs associated with QST use, which included paying for broadband data, subscriptions, or device upgrades. On the other hand, there were patients with diabetes who recognised that QST was beneficial for diabetic food selection, which could save money. In this respect, participants P12 and P17 noted,

*“I frequently find it challenging to afford nutritious foods, resulting in a poor diet. I cannot afford to fund technological use for my diabetes, I prioritise food. Worse of, I earn very little money that cannot meet my budgetary requirements.”*

(P12)

*“The funds I possess are designated for my family’s sustenance. They say this technology will benefit me in the long term, yet how can I concern myself with the future when I am uncertain about my ability to eat today? The technology does not simplify my life; it merely introduces an additional expense I cannot bear.”*

(P17)

Hence, the results uncovered that immediate food subsistence needs shaped how some of the patients with diabetes set their financial preferences with respect to the use of QST. Even where potential long-term health benefits were acknowledged, short-term financial realities influenced spending decisions.

#### 5.3.2. Employment

Unemployment negatively shaped participants’ ability to maintain consistent engagement with QST. Technology-related expenses were frequently described as discretionary rather than essential when compared with immediate living costs. In the absence of stable income, technology-related costs were often deprioritised in favour of essential expenditures such as food, medication, and household needs. There were patients with diabetes who disclosed that they depended on family support, which introduced additional uncertainty with regard to the sustainable use of QST for diabetes management. In this respect, participants P1 and P18 said,

*“My family supports me by sending money so that I can buy data bundles. I cannot afford my medications or the necessary technology. Although their efforts to send me money to keep me afloat, I divert the funds to cover other expenses since I’m unemployed.”*

(P1)

*“I am a student and unemployed. Every cent is significant. Managing my diabetes has become my least priority. My primary concerns revolve around procuring my next meal and university requirements.”*

(P18)

The findings indicate that unemployment limits access to digital health tools, hence undermining self-care practices. Consequently, the potential health-promoting benefits of QST may remain inaccessible to unemployed individuals, thereby reinforcing socioeconomic inequities in chronic disease management.

The results of this study were discussed under three themes, which are technology investment cost, conventional healthcare cost, and socio-economic constraints. The themes are discussed in the following section.

## 6. Discussion

The research question answered was, “How do patients with diabetes perceive and experience the economic burden of using QST in Zimbabwe?” Data analysis uncovered three themes that pertain to how patients with diabetes experience the costs associated with the use of QST in managing diabetes. The themes are technology investment cost, conventional health care cost, and socio-economic constraints. The sections that follow discuss these themes.

### 6.1. Technology Investment Cost

The technology investment cost pertains to the expenses of acquiring, implementing, and maintaining the use of QST for managing diabetes. The results of this study suggested that the technology investment cost of acquiring QST outweighed the benefits of self-monitoring diabetes amongst many patients with diabetes. The technology investment cost covers the procurement of QST-compatible devices, broadband data cost, subscription fees, electricity(power) supply, and technical support cost. Even though the patients with diabetes acknowledged the benefits of QST in the bigger picture of their health management, they perceived the investment cost as beyond their reach. The findings of this study should be understood in the context of low-income countries because they reflect the realities of a third-world country in Southern Africa. The findings of this study resonate with previous literature, which suggests that high initial costs can be a hindrance to the adoption and effective use of health technologies [51,52,53]. There is literature that aligns with the findings of this study, which confirms that in low- and middle-income countries, investment costs are barriers to equitable implementation of technology [25,53].

This study uncovered that although individuals may struggle with investment costs, the costs were absorbed by others, for example, an employer, family or a community. There were participants who disclosed that they benefited from a WiFi connection at work, and there were participants who acknowledged that they received financial donations to fund QST from family members. Therefore, the technology investment cost can be absorbed by others, which helps individuals with limited resources.

While prior literature reports potential long-term efficiencies associated with QST in technologically resourced contexts [54], participants in this study were responsible for initial and recurring technology costs. This indicates that technology investment overhead differs substantially between instances where technology access is privately financed and where it is institutionally supported. Nevertheless, the literature sometimes does not recognise that the initial out-of-pocket costs are solely on the patient, which is prevalent in the context of developing countries [55]. Hence, the findings of this study suggest that patients with diabetes may need health insurance with an initial technology investment to acquire devices, pay for internet, and pay subscription fees.

Theoretically, these findings reflect the importance of facilitating conditions and perceived behavioural control in shaping technology adoption and sustained use. In line with UTAUT, high device costs, broadband expenses, and subscription fees undermine facilitating conditions, thereby constraining long-term adoption. Similarly, within the Theory of Planned Behaviour, patients with diabetes perceived behavioural control is weakened by financial and infrastructural barriers, limiting their ability to translate positive attitudes towards QST into sustained behavioural change. From a Social Cognitive Theory perspective, low self-efficacy stemming from financial insecurity further diminishes patients’ confidence in their ability to manage their condition using technology.

### 6.2. Conventional Healthcare Cost

The results revealed that diabetes, as a chronic disease, requires patients to follow conventional health practices, which require physician consultation fees. The study found that once patients understood QST’s role in overall diabetes management, they recognised its long-term benefits but not its economic limitations. The diabetes patients assumed that QST would significantly reduce their medical costs in the short run, but this was not true, because they still had to depend on costly conventional healthcare. The patients with diabetes had to consult with a physician, do laboratory blood tests and obtain medical prescriptions.

This study offers a different perspective from existing literature, which suggests that incorporating QST into daily practice may reduce medical consultations and related costs. This study showed that while QST provides valuable information, it cannot replace expert medical advice and support. This aligns with literature that found that technology cannot fulfil all health requirements for managing chronic diseases [43,56]. Thus, QST’s short-term measurable advantages in cost-saving were not appreciated by the patients with diabetes in this study. QST served as an auxiliary instrument rather than a cost-reducing alternative. This aligns with literature that argues that QST complements, rather than substitutes, professional clinical services in chronic disease management [43,56]. The findings of this study provide empirical evidence to support the argument that there is an economic misconception that implementing QSTs in health will reduce costs [42].

### 6.3. Socio-Economic Costs

The socio-economic status of patients with diabetes determined their readiness to use QST for managing diabetes. The results identified disparities in health investment priorities between patients with diabetes from lower-income and upper-income families. For those from lower-income families, cost was a barrier, and they prioritised spending their finances on day-to-day living expenses over QST. Consistent with the literature on household financial burdens [57,58], essential expenses such as food and medication were frequently prioritised over digital health costs. In such contexts, technology-related expenses were often framed as discretionary rather than essential, even when perceived as beneficial. These findings suggest that economic vulnerability influences how participants prioritised and sustained engagement with QST, particularly where income instability limited flexibility in household budgeting. The results of this study confirm the cost-driven underuse of QST by patients with diabetes from lower-income families [59].

## 7. Implications

The findings of this study offer several considerations for digital health research and practice in resource-constrained settings. First, the economic experience of QST adoption appears to extend beyond device affordability to include recurring connectivity costs, infrastructural reliability, and household-level financial priorities. This suggests that evaluations of digital self-management tools may benefit from incorporating contextual affordability assessments rather than focusing solely on clinical or technical performance metrics.

Second, the persistence of conventional healthcare expenditures alongside QST use indicates that digital monitoring tools are often experienced as complementary rather than substitutive within existing care routines. This observation aligns with prior research suggesting that digital health adoption does not automatically translate into reduced system-level utilisation in low-income country contexts. Future investigations may therefore consider examining how digital tools are integrated into existing workflows rather than assuming the displacement of traditional services.

Third, socio-economic vulnerability emerged as an influential contextual factor shaping sustained engagement. While this study does not evaluate specific design strategies or policy interventions, the findings underscore the importance of situating digital health deployment within the material realities of connectivity access, electricity reliability, and income stability. Attention to these contextual dimensions may enhance the interpretive relevance of digital health initiatives across similar settings.

Finally, this study contributes qualitative evidence to ongoing discussions on the affordability of digital health in public health from a third-world country. By foregrounding patient-level cost experiences, it complements economic modelling studies and highlights the value of incorporating experiential data into broader assessments of digital health implementation.

## 8. Conclusions, Limitations and Future Works

This study provides qualitative, patient-centred insight into how individuals with diabetes experience the costs associated with adopting quantified self-technologies in a third-world country, specifically, the case of Zimbabwe. Participants described layered financial considerations extending beyond device acquisition to include recurring digital expenses, infrastructural constraints, and continued reliance on conventional healthcare services. These findings indicate that the economic implications of QST adoption are context-sensitive and shaped by local health system structures and household financial realities. Rather than advancing definitive economic claims, this study provides experiential evidence that complements formal cost-effectiveness research and underscores the importance of situating digital health affordability within specific socioeconomic contexts.

This study has several limitations that should be considered when interpreting the findings. First, the research employed a qualitative exploratory design with a purposive sample of 20 patients with diabetes in Zimbabwe. While this approach enabled rich, in-depth insights into patient experiences, the findings are not statistically generalizable to the broader population of patients with diabetes. Second, the study focused exclusively on patient-centred perspectives to foreground lived cost experiences, which are often underrepresented in digital health evaluations. However, the absence of clinician, policymaker, and technology developer perspectives limits the ability to draw system-level economic conclusions. Future studies should adopt multi-stakeholder or mixed-method approaches to triangulate patient experiences with clinical workflows, policy priorities, and health system cost structures. Third, the study relied on self-reported cost experiences, which may be subject to recall bias or subjective interpretation of financial burden. Although the findings are grounded in Zimbabwe’s specific health and economic context, many of the structural constraints identified (out-of-pocket financing, infrastructure instability) may resonate across other low-resource health systems; nevertheless, transferability should be assessed case-by-case.

Future work may conduct a quantitative cost–benefit analysis to examine comparative healthcare expenditure patterns and compare the actual healthcare expenses for QST users and non-users over an extended period. Furthermore, future studies could also explore the provider perspective through the evaluation of the patient-experience cost burden and usefulness of such technologies in the management of chronic illnesses, such as diabetes, in a given setting, such as that of Zimbabwe. It would also be important for future research to examine the cost implications and outcomes that result from the implementation of QST among chronic illness sufferers in low-income countries through longitudinal research, which would offer important insights through comparative analyses.

## Figures and Tables

**Table 1:** Mapping of interview questions to theoretical framework constructs.

Interview Question	Study Focus	TPB Construct	UTAUT Construct	SCT Construct
Q1. Could you describe your journey with diabetes management and the challenges you face?	Baseline disease management context and lived experience	Background beliefs	—	Environmental determinants; reciprocal determinism
Q2. Do you believe mobile health applications made your diabetes management more affordable? Why or why not?	Perceived economic impact of QST adoption	Attitude toward behaviour	Performance expectancy	Outcome expectations
Q3. What resources do you have to help you use mobile health applications?	Access to devices, data, electricity, skills, and support	Perceived behavioural control	Facilitating conditions	Environmental determinants
Q4. Can you detail the various healthcare costs you incur for your diabetes management?	Baseline healthcare cost structure	Perceived behavioural control	—	Environmental constraints
Q5. What are the direct, indirect and unexpected costs of using your mobile health applications?	Digital health cost ecosystem	Perceived behavioural control	Facilitating conditions	Environmental constraints
Q6. What were your initial expectations regarding using this technology, especially concerning its impact on your healthcare costs?	Technology optimism and cost-saving beliefs	Attitude toward behaviour	Performance expectancy	Outcome expectations
Q7. How has the use of mobile health applications affected these costs, if at all?	Post-adoption economic outcomes	Attitude revision	Realised performance expectancy	Experiential learning
Q8. How does the Zimbabwean healthcare system and economic context influence your ability to manage diabetes and use mobile health applications?	Health system and socio-economic context	Perceived behavioural control	Facilitating conditions	Environmental determinants
Q9. What advice would you give to other patients with diabetes considering using mobile health applications in Zimbabwe?	Peer learning and social influence	Subjective norms	Social influence	Observational learning

The interview guide was explicitly designed to operationalise the core constructs of the TPB, the UTAUT and SCT, thereby ensuring conceptual coherence between the theoretical framework, data collection instrument, and analytical strategy.

## Data Availability

The data presented in this study are available on request from the corresponding author due to privacy and ethical reasons.

## Abbreviations

The following abbreviations are used in this manuscript:

QST	Quantified Self Technology
CGM	Continuous Glucose Monitor
NCD	Non-communicable Disease
LMICs	Low to middle-income countries

## References

[1] Scheidt-Nave C., Heidemann C., Reitzle L., Buchmann M., Ziese T., Icks A. (2024). Diabetes surveillance—Laying the groundwork for non-communicable dis ease surveillance in Germany. J. Health Monit..

[2] Butt M.D., Ong S.C., Rafiq A., Kalam M.N., Sajjad A., Abdullah M., Malik T., Yaseen F., Babar Z.U.D. (2024). A systematic review of the economic burden of diabetes mellitus: Contrasting perspectives from high and low middle-income countries. J. Pharm. Policy Pract..

[3] Sun H., Saeedi P., Karuranga S., Pinkepank M., Ogurtsova K., Duncan B.B., Stein C., Basit A., Chan J.C.N., Mbanya J.C. (2022). IDF Diabetes Atlas: Global, regional and country-level diabetes prevalence estimates for 2021 and projections for 2045. Diabetes Res. Clin. Pract..

[4] Li X. (2025). The Research of Influence Factors and Preventive Measures that Possibly lead to Diabetes. Theor. Nat. Sci..

[5] Ratre B., Patel D., Patel D., Patel H., Patel S., Singh D. (2024). Review Paper for Diabetes Mellitus. Int. J. Adv. Multidiscip. Res. Stud..

[6] Wang Y., Zhang P., Shao H., Andes L.J., Imperatore G. (2022). Medical Costs Associated with Diabetes Complications in Medicare Benef iciaries Aged 65 Years or Older with Type 1 Diabetes. Diabetes Care.

[7] Da Ros R., Assaloni R., Michelli A., Brunato B., Barro E., Meloni M., Miranda C. (2024). Burden of Infected Diabetic Foot Ulcers on Hospital Admissions and Costs in a Third-Level Center. Diabetology.

[8] Boateng D., Ayellah B.B., Adjei D.N., Agyemang C. (2022). Contribution of diabetes to amputations in sub-Sahara Africa: A systematic review and meta-analysis. Prim. Care Diabetes.

[9] Mutowo M.P., Lorgelly P.K., Laxy M., Renzaho A.M.N., Mangwiro J.C., Owen A.J. (2016). The hospitalization costs of diabetes and hypertension complications in Zimbabwe: Estimations and correlations. J. Diabetes Res..

[10] Mutyambizi C., Pavlova M., Chola L., Hongoro C., Groot W. (2018). Cost of diabetes mellitus in Africa: A systematic review of existing literature. Global. Health.

[11] Moucheraud C., Lenz C., Latkovic M., Wirtz V.J. (2019). The costs of diabetes treatment in low- and middle-income countries: A systematic review. BMJ Glob. Health.

[12] Kangas T., Ayala R. (2023). Continuous Glucose Monitoring—Offering empowerment and self-care agency for type 1 diabetes patients. Salud Cienc. Tecnol..

[13] Ndlovu B.M., Chipangura B., Singh S. (2025). The readiness to use quantified self-technology: A case of diabetic patients from a hospital in Bulawayo, Zimbabwe. Digit. Health.

[14] Adeghe E.P., Okolo C.A., Ojeyinka O.T. (2024). A review of wearable technology in healthcare: Monitoring patient health and enhancing outcomes. Open Access Res. J. Multidiscip. Stud..

[15] Huang E.S., Sinclair A., Conlin P.R., Cukierman-Yaffe T., Hirsch I.B., Huisingh-Scheetz M., Kahkoska A.R., Laffel L., Lee A.K., Lee S. (2023). The Growing Role of Technology in the Care of Older Adults with Diabet es. Diabetes Care.

[16] Mutunhu B., Chipangura B., Singh S. (2024). An Exploration of Opportunities for Quantified-Self Technology in Diabetes Self-Care: A Systematic Literature Review. J. Health Inform. Afr..

[17] Tortorella G.L., Fogliatto F.S., Espôsto K.F., Mac Cawley A.F., Vassolo R., Tlapa D., Narayanamurthy G. (2022). Healthcare costs’ reduction through the integration of Healthcare 4.0 technologies in developing economies. Total Qual. Manag. Bus. Excell..

[18] Religioni U., Barrios-Rodríguez R., Requena P., Borowska M., Ostrowski J. (2025). Enhancing Therapy Adherence: Impact on Clinical Outcomes, Healthcare Costs, and Patient Quality of Life. Medicina.

[19] Khan N., Marvel F.A., Wang J., Martin S.S. (2017). Digital Health Technologies to Promote Lifestyle Change and Adherence. Curr. Treat. Options Cardiovasc. Med..

[20] Sutarno M., Anam K. (2022). AnEmpiricalStudyontheUseofDigitalTechnologies to Achieve Cost-Effectiveness in Healthcare Management. Am. J. Health Behav..

[21] Thirunavukkarasu A., Alsaidan A.A. (2025). Self-Management Behaviours in Type 2 Diabetes Across Gulf Cooperation Council Countries: An Updated Narrative Review to Enhance Patient Care. Healthcare.

[22] Miranda J.C., Raza S.A., Kolawole B., Khan J.K., Alvi A., Ali F.S., Chukwudi E.E., Ram N., Oluwatoyin A. (2023). Enhancing Diabetes Care in LMICs: Insights from a Multinational Consensus. Pak. J. Med. Sci..

[23] Njagi P., Groot W., Arsenijevic J., Dyer S., Mburu G., Kiarie J. (2023). Financial costs of assisted reproductive technology for patients in low-and middle-income countries: A systematic review. Hum. Reprod. Open.

[24] Merchant R., Torous J., Rodriguez-Villa E., Naslund J.A. (2020). Digital technology for management of severe mental disorders in low-income and middle-income countries. Curr. Opin. Psychiatry.

[25] Dhyani V.S., Krishnan J.B., Mathias E.G., Hossain M.M., Price C., Gudi N., Pattanshetty S., Zodpey S. (2023). Barriers and facilitators for the adoption of telemedicine services in low-income and middle-income countries: A rapid overview of reviews. BMJ Innov..

[26] Munetsi D., Maphosa F. (2024). Rethinking Healthcare Democracy in the Era of Digital Technologies: Insights from Hard-to-Reach Communities in Southern Zimbabwean Provinces.

[27] Batani J., Maharaj M.S. (2025). Emerging technologies’ role in reducing under-five mortality in a low-resource setting: Challenges and perceived opportunities by public health workers in Makonde District, Zimbabwe. J. Child Health Care.

[28] Mtshali S., Hongoro C., Mahomed O. (2022). Hospitalization Costs for Diabetes Related Lower Extremity Amputation at a Referral Hospital in KwaZulu Natal, South Africa. Curr. Diabetes Rev..

[29] Adamjee E., de Harerimana J.D. (2022). Estimating the Economic Burden of Diabetes Mellitus in Kenya: A Cost of Illness Study. Eur. Sci. J. ESJ.

[30] Bober T.M.M., Krall J.S.S., Garvin S., Zupa M., White C., Sotaa B., Low C., Rosland A.-M. (2024). 564-P: Insights into How Health Technologies Can Support Patient-Centered Diabetes Self-Management—A Mixed-Methods Approach. Diabetes.

[31] Shafeeq D.M., Khan D.Z. (2023). Digital health interventions for diabetes self-management: Harnessing technology for improved outcomes. Int. J. Diabetes Res..

[32] Dwibedi C., Mellergård E., Gyllensten A.C., Nilsson K., Axelsson A.S., Bäckman M., Sahlgren M., Friend S.H., Persson S., Franzén S. (2022). Effect of self-managed lifestyle treatment on glycemic control in patients with type 2 diabetes. NPJ Digit. Med..

[33] Aggarwal A., Pathak S., Goyal R. (2022). Clinical and economic outcomes of continuous glucose monitoring system (CGMS) in patients with diabetes mellitus: A systematic literature review. Diabetes Res. Clin. Pract..

[34] Uhl S., Choure A., Rouse B., Loblack A., Reaven P. (2024). Effectiveness of Continuous Glucose Monitoring on Metrics of Glycemic Control in Type 2 Diabetes Mellitus: A Systematic Review and Meta-analysis of Randomized Controlled Trials. J. Clin. Endocrinol. Metab..

[35] Edo O.C., Ang D., Etu E.-E., Tenebe I., Edo S., Diekola O.A. (2023). Why do healthcare workers adopt digital health technologies—A cross-sectional study integrating the TAM and UTAUT model in a developing ec onomy. Int. J. Inf. Manag. Data Insights.

[36] Bertolazzi A., Quaglia V., Bongelli R. (2024). Barriers and facilitators to health technology adoption by older adults with chronic diseases: An integrative systematic review. BMC Public Health.

[37] Bloss C.S., Wineinger N.E., Peters M., Boeldt D.L., Ariniello L., Kim J.Y., Sheard J., Komatireddy R., Barrett P., Topol E.J. (2016). A prospective randomized trial examining health care utilization in individuals using multiple smartphone-enabled biosensors. PeerJ.

[38] Yegros-Yegros A., van de Klippe W., Abad-Garcia M.F., Rafols I. (2020). Exploring why global health needs are unmet by research efforts: The potential influences of geography, industry and publication incentives. Health Res. Policy Syst..

[39] Okpala P. (2018). Assessment of the influence of technology on the cost of healthcare service and patient’s satisfaction. Int. J. Healthc. Manag..

[40] Chang H., Choi J.Y., Shim J., Kim M., Choi M. (2023). Benefits of Information Technology in Healthcare: Artificial Intelligence, Internet of Things, and Personal Health Records. Healthc. Inform. Res..

[41] LaMarca A., Tse I., Keysor J. (2023). Rehabilitation Technologies for Chronic Conditions: Will We Sink or Swim?. Healthcare.

[42] Feng S., Mäntymäki M., Dhir A., Salmela H. (2021). How Self-tracking and the Quantified Self Promote Health and Well-bein g: Systematic Review. J. Med. Internet Res..

[43] Samal L., Fu H.N., Camara D.S., Wang J., Bierman A.S., Dorr D.A. (2021). Health information technology to improve care for people with multiple chronic conditions. Health Serv. Res..

[44] Ajzen I. (1991). The theory of planned behavior. Organ. Behav. Hum. Decis. Process..

[45] Venkatesh V., Morris M.G., Davis G.B., Davis F.D. (2003). User acceptance of information technology: Toward a unified view. MIS Q. Manag. Inf. Syst..

[46] Bandura A. (1986). Social Foundations of Thought and Action: A Social Cognitive Theory.

[47] Walliman N. (2021). Research Theory.

[48] Wang X. (2024). Use of proper sampling techniques to research studies. Appl. Comput. Eng..

[49] Cresswell J., Creswell J. (2018). Research Design: Qualitative, Quantitative, and Mixed Methods Approaches.

[50] Braun V., Clarke V. (2012). Thematic analysis. APA Handbook of Research Methods in Psychology, Vol. 2. Research Designs: Quantitative, Qualitative, Neuropsychological, and Biological.

[51] Addala A., Suttiratana S.C., Wong J.J., Lanning M.S., Barnard K.D., Weissberg-Benchell J., Laffel L.M., Hood K.K., Naranjo D. (2021). Cost considerations for adoption of diabetes technology are pervasive: A qualitative study of persons living with type 1 diabetes and their families. Diabet. Med..

[52] Agarwal S., Simmonds I., Myers A.K. (2022). The Use of Diabetes Technology to Address Inequity in Health Outcomes: Limitations and Opportunities. Curr. Diab. Rep..

[53] Kazibwe J., Tran P.B., Annerstedt K.S. (2021). The household financial burden of non-communicable diseases in low- and middle-income countries: A systematic review. Health Res. Policy Syst..

[54] Wan W., Skandari M.R., Minc A., Nathan A.G., Winn A., Zarei P., O’Grady M., Huang E.S. (2018). Cost-effectiveness of Continuous Glucose Monitoring for Adults with Type 1 Diabetes Compared with Self-Monitoring of Blood Glucose: The DIAMOND Randomized Trial. Diabetes Care.

[55] Gupta Y., Goyal A., Tandon N. (2023). Implications of technology guidelines for low-income and middle-income countries. Lancet Diabetes Endocrinol..

[56] Taylor M.L., Thomas E.E., Vitangcol K., Marx W., Campbell K.L., Caffery L.J., Haydon H.M., Smith A.C., Kelly J.T. (2022). Digital health experiences reported in chronic disease management: An umbrella review of qualitative studies. J. Telemed. Telecare.

[57] Monaco A., Palmer K., Faber N.H.R., Kohler I., Silva M., Vatland A., van Griensven J., Votta M., Walsh D., Clay V. (2021). Digital health tools for managing noncommunicable diseases during and after the COVID-19 pandemic: Perspectives of patients and caregivers. J. Med. Internet Res..

[58] Choy M.A., O’Brien K., Barnes K., Sturgiss E.A., Rieger E., Douglas K., He G.C. (2024). Evaluating the Digital Health Experience for Patients in Primary Care: Mixed Methods Study. J. Med. Internet Res..

[59] Alkabbani W., Cromer S.J., Kim D.H., Paik J.M., Bykov K., Munshi M., Wexler D.J., Patorno E. (2025). Overall Uptake and Racial, Ethnic, and Socioeconomic Disparities in the Use of Continuous Glucose Monitoring Devices Among Insulin-Treated Older Adults with Type 2 Diabetes. Diabetes Care.

